# Isocitrate dehydrogenase 1 gene mutations: a case review unveiling its biological impact on disease progression, prognosis and treatment in Chilean patients

**DOI:** 10.1093/bjrcr/uaaf019

**Published:** 2025-03-24

**Authors:** Tomás de Mayo Glasser, Benjamín García-Bloj, Juan A Godoy, Fernando Sigler Chávez, Ignacio N Retamal, Fernán Gómez-Valenzuela, Ian Silva, Matías Muñoz-Medel, Carolina Sánchez, Felipe Pinto, Paola Aravena, Ignacio Corvalán, José M Erpel, Patricio A Manque, Marcelo Garrido

**Affiliations:** Centro de Oncología de Precisión, Facultad de Medicina y Ciencias de la Salud, Universidad Mayor, Santiago 7500000, Chile; Centro de Oncología de Precisión, Facultad de Medicina y Ciencias de la Salud, Universidad Mayor, Santiago 7500000, Chile; Centro de Oncología de Precisión, Facultad de Medicina y Ciencias de la Salud, Universidad Mayor, Santiago 7500000, Chile; Centro de Oncología de Precisión, Facultad de Medicina y Ciencias de la Salud, Universidad Mayor, Santiago 7500000, Chile; Centro de Oncología de Precisión, Facultad de Medicina y Ciencias de la Salud, Universidad Mayor, Santiago 7500000, Chile; Centro de Oncología de Precisión, Facultad de Medicina y Ciencias de la Salud, Universidad Mayor, Santiago 7500000, Chile; Centro de Oncología de Precisión, Facultad de Medicina y Ciencias de la Salud, Universidad Mayor, Santiago 7500000, Chile; Centro de Oncología de Precisión, Facultad de Medicina y Ciencias de la Salud, Universidad Mayor, Santiago 7500000, Chile; Centro de Oncología de Precisión, Facultad de Medicina y Ciencias de la Salud, Universidad Mayor, Santiago 7500000, Chile; Centro de Oncología de Precisión, Facultad de Medicina y Ciencias de la Salud, Universidad Mayor, Santiago 7500000, Chile; Centro de Oncología de Precisión, Facultad de Medicina y Ciencias de la Salud, Universidad Mayor, Santiago 7500000, Chile; Centro de Oncología de Precisión, Facultad de Medicina y Ciencias de la Salud, Universidad Mayor, Santiago 7500000, Chile; Centro de Oncología de Precisión, Facultad de Medicina y Ciencias de la Salud, Universidad Mayor, Santiago 7500000, Chile; Centro de Oncología de Precisión, Facultad de Medicina y Ciencias de la Salud, Universidad Mayor, Santiago 7500000, Chile; Center for Genomics and Bioinformatics (CGB), Faculty of Science, Universidad Mayor, Santiago 7500000, Chile; Centro de Oncología de Precisión, Facultad de Medicina y Ciencias de la Salud, Universidad Mayor, Santiago 7500000, Chile

**Keywords:** IDH1, oncometabolite, TP53

## Abstract

Isocitrate dehydrogenase 1 gene (*IDH1*, [NADP (+)] 1) encodes for an enzyme that catalyses the oxidative decarboxylation of isocitrate into α-ketoglutarate. However, it is well known that mutant *IDH1* (mu/*IDH*1) promotes the accumulation of D2-hydroxyglutarate, an oncometabolite that stimulates tumourigenesis through various secondary, complex metabolic effects. *IDH1* and also *IDH2* gene mutations have been identified in several types of cancers, such as gliomas, conventional central and periosteal malignant cartilaginous tumours, cytogenetically normal acute myeloid leukaemia, and cholangiocarcinoma. Here, we present 4 cases of Chilean patients with different primary malignant tumours harbouring *IDH1*. One patient carried the *IDH1* p. R132H mutation, the other has *IDH1* p. R132L mutation, and the last 2, *IDH1* p. R132C mutation. Of note, all these patients had a very poor response to chemotherapy and a rapid disease progression, resulting in a relatively swift death. Next-Generation Sequencing results highlighting mutations in those genes, and other cancer genes were further subjected to *in silico* study of protein-protein interactions, gene ontology, and pathway enrichment. We also include a state-of-the-art literature review about *IDH1* and *IDH2* molecular biology, biochemical properties, and the role of their mutations in cancer development and progression, along with insights into regional variations in cancer biology and treatment response.

## Introduction

Isocitrate dehydrogenase (IDH) is a vital enzyme in the tricarboxylic acid cycle, present in various organisms. Both IDH1 and IDH2 catalyse the oxidative decarboxylation of isocitrate to α-ketoglutarate (α-KG), but they have distinct roles and cellular localization: IDH1 is cytoplasmic, whereas IDH2 is mitochondrial.^1^ Research on solid tumours, a pan-cancer analysis, has demonstrated that *IDH1* mutations occur more frequently and affect a broader range of cancer types compared to *IDH2*. In a study involving 5149 patients, 205 *IDH* mutations (3.78%) were identified, including 145 *IDH1* mutations (2.68%) and 63 *IDH2* mutations (1.16%).^2^

**Figure 1.**
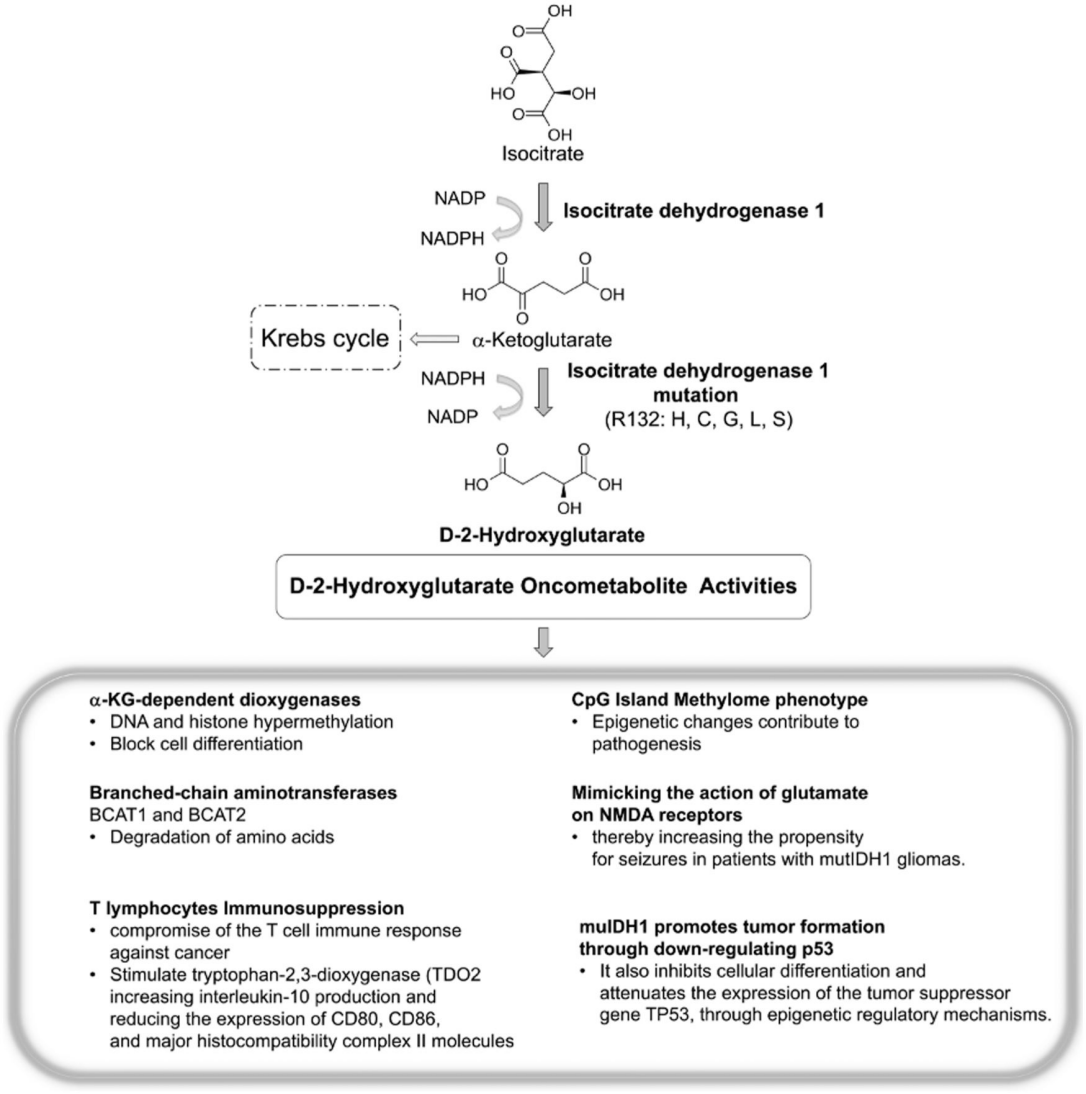
Mutant *IDH1* catalyse the production of D-2-hydroxyglutarate (2HG) from α-keto-glutarate (αKG). Cancer-associated *IDH1* mutations confer a gain of function that promotes the production of D-2HG, an oncometabolite that facilitates neoplastic transformation. The accumulation of D-2HG inhibits multiple αKG-dependent dioxygenases involved in various cellular and biological pathways. Variants such as R132H, R132C, R132G, R132L, and R132S have been identified, all of which produce D-2HG and exert inhibitory effects on cellular metabolism. C = cysteine; G = glycine; H = histidine; L = leucine; NADP = nicotinamide adenine dinucleotide phosphate; NADPH = nicotinamide adenine dinucleotide phosphate hydrogen, R = arginine; S = serine.

IDH enzymes operate as homodimers using reduced nicotinamide adenine dinucleotide phosphate (NADP+) as a coenzyme for electron transfer. Each dimer consists of 2 asymmetric monomers with 3 structural domains, one of which is significantly larger. The wild-type IDH enzyme catalyses the conversion of cytosolic isocitrate to α-KG, carbon dioxide, and nicotinamide adenine dinucleotide phosphate hydrogen (NADPH) through NADP^+^-dependent oxidative decarboxylation.^3–5^ These mutations are generally somatic, heterozygous, and occur at specific arginine residues, including *IDH1* R132, IDH2 R140, or R172^6^ (Figure 1).

The IDH1 mutation is the substitution of arginine 132 by other amino acids, including R132 H/C/L/S.^7^ Particularly, R132H and R132C are the most common mutation types, with mutation’s frequency ranging from 4.3% to 33%.^8^^,^^9^

MuIDH1/2 enzymes produce D2-hydroxyglutarate (D-2HG), a competitive inhibitor of several α-KG-dependent dioxygenases, including histone demethylases and the TET family of 5-methylcytosine hydroxylases. By occupying the α-KG binding site in histone demethylases, D-2HG acts as an α-KG antagonist. As a result, tumour-derived mIDH1 and mIDH2 lead to the accumulation of D-2HG, resulting in genome-wide alterations in histone and DNA methylation.^10^ muIDH1 protein functions as an oncogene, and D-2HG interacts with additional proteins implicated in oncogenesis.^11^

## Molecular effects of IDH mutations

Of note, D-2HG has a strong structural resemblance to α-KG, which means that it competes with it within the cellular environment, and thus, impairing α-KG binding to its traditional substrates, leading to the inhibition of α-KG-dependent dioxygenases,^10^ such as TET-DNA demethylases and Jumonji family histone demethylases (KDMs), resulting in DNA and histone hypermethylation and a block in cell differentiation.^12^ Additionally, collagen propyl-4-hydroxylase (PHD) reduced collagen hydroxylation^13^ and hypoxia-inducing factor (HIF) PHD inhibition leads to elevated levels of HIF-1α, and PHD regulates the stability of HIF-1α. Under normoxic conditions, PHD hydroxylates proline residues in HIF-1α, leading to its ubiquitination and subsequent proteasomal degradation. Conversely, under hypoxic conditions, hydroxylation is inhibited, resulting in the accumulation of HIF-1α,^14^ and indirectly activates the HIF-1α pathway.^15^ In addition, D-2HG affects the activity of transaminases, including branched-chain aminotransferases, BCAT1 and BCAT2, which are crucial for the degradation of branched amino acids.^16^ As noted above, D-2HG also impacts immunoregulatory functions in tumour activity, as it is released by tumour cells in the interstitial space within the tumour microenvironment, causing it to accumulate and then taken up by T lymphocytes, ultimately compromising their capacity to mediate anticancer immune responses, impairing CD8^+^ T-cell proliferation and cytotoxic capacity.^17–20^ D-2HG can stimulate tryptophan 2,3-dioxygenase (TDO_2_) activity in macrophages, thereby promoting tryptophan metabolism and kynurenine production. This metabolite induces the translocation of the aryl hydrocarbon receptor to the nucleus, consequently increasing interleukin-10 production and reducing the expression of CD80, CD86, and major histocompatibility complex II molecules.^21^ These alterations result in decreased antigen presentation and enhanced T-cell inhibition, thus contributing to a more immunosuppressive tumour microenvironment. Furthermore, D-2HG can transiently induce hypermethylation of the programmed cell death ligand 1 (PD-L1) promoter, subsequently leading to reduced PD-L1 expression.^22^ mu/*IDH1* has been shown to play a role in the remodelling of the methylome in gliomas. Specifically, *IDH1* establishes the CpG island methylator phenotype, which is characterized by extensive epigenetic changes that contribute to tumour pathogenesis.^23^^,^^24^ In this milieu, D-2HG produced by mu/*IDH1* may enhance neuronal activity by mimicking the action of glutamate on NMDA receptors, thereby increasing the propensity for seizures in patients with mu/*IDH1* gliomas. This finding has significant translational implications for the personalized management of tumour-associated epilepsy, as targeted mu/*IDH1* inhibitors could potentially ameliorate antiepileptic treatment in patients with mu/*IDH1* gliomas (Figure 1).

Regarding our case reviews, a series of 4 patients with diverse cancer types, all exhibiting mutations in the *IDH1* gene are presented. One patient demonstrated an IDH1 R132H mutation, another exhibited an R132L mutation, and 2 patients manifested R132C mutations. The Molecular Tumor Board of the COP-U Mayor (MTB-COP U Mayor) analysed these cases to inform future clinical decisions regarding the treatment of patients with IDH mutations, based on the findings observed in Chilean cancer patients cohorts. Enhanced comprehension and knowledge of these mutations, specifically elucidating variables such as genetic epidemiology and the biological impact of different *IDH1/IDH2* mutation types in Chilean patients, may be crucial for advancing personalized oncology approaches in our country, potentially guiding more targeted therapies, and improving patient outcomes. Furthermore, this research could contribute valuable insights into regional variations in cancer biology and treatment response.

## Materials and methods

All patients were referred to MTB-COP U Mayor for a comprehensive analysis of the molecular characteristics of their mutations. Clinical characteristics, laboratory findings, and radiological assessments were obtained from medical records. Tumoural DNA extracted from Formalin-Fixed Paraffin-Embedded tissue or ctDNA obtained from plasma samples for Liquid Biopsy was analysed using Next-Generation Sequencing (NGS) for cancer-related genes in selected patients with primary malignancies.

### Next-generation sequencing

Samples were analysed using NGS services (Foundation One Liquid). Next-Generation Sequencing was performed using a panel of 364 cancer-related genes (Foundation Medicine; Roche, USA).

### Genetic mutation analysis

Mutational profiles were analysed based on the expression of IDH1 and associated gene mutations. Mutation frequency and type for each case were compiled into tabular datasets using the “tibble” R package (v4.3.0). Each dataset included *IDH1* and patient-specific genetic alterations.

### Protein-protein interaction analysis

To assess protein-protein interactions, the STRING database (v12.0) was utilized. The mutations were mapped to STRING identifiers using the “STRING db” R package. High-confidence interactions (score threshold ≥400) were visualized using the “igraph” package. Interaction networks were simplified by removing loops and multiple edges, and node labels were assigned based on common gene symbols.

### Gene ontology enrichment

The Gene Ontology (GO) enrichment analysis was conducted using the “cluster Profiler” R package. Functional enrichment for biological processes, molecular functions, and cellular components was performed using enrich GO. The Benjamini-Hochberg method was applied for *P*-value adjustment, with a q-value cutoff of 0.05. Results were visualized through dot plots.

### Kyoto Encyclopedia of Genes and Genomes pathway analysis

Pathway enrichment was performed using the Kyoto Encyclopedia of Genes and Genomes (KEGG) via enrich KEGG. Significant pathways (q-value < 0.05) were plotted using the dot plot function from the “enrichplot” R package. Visualization parameters were customized to highlight the key pathways and gene interactions relevant to each clinical case.

Written informed consent was obtained from the patient(s) for publication of this case review.

## Results

### Case presentation of patients with IDH1 mutations

#### Patient 1

Patient History: Right frontal tumour of dysembryonic aetiology.

Diagnosis: Oligodendroglioma relapse surgically excised in 2009. Secondary epilepsy, currently managed with Levetiracetam.

Microsatellite status: MS-stable.

Tumour Mutational Burden (TMB): 4 Muts/Mb.

#### Identification of genomic alterations

Isocitrate Dehydrogenase (NADP (+)) 1, (*IDH1*, R132H); Methylthioadenosine Phosphorylase (*MTAP*, loss); Cyclin Dependent Kinase Inhibitor 2A/2B (*CDKN2A/B*, loss); Nibrin (*NBN*, K544*); Neurotrophic Receptor Tyrosine Kinase 1 (*NTRK1*, amplification); *TP53* (R273C).

#### VUS

**Figure 2.**
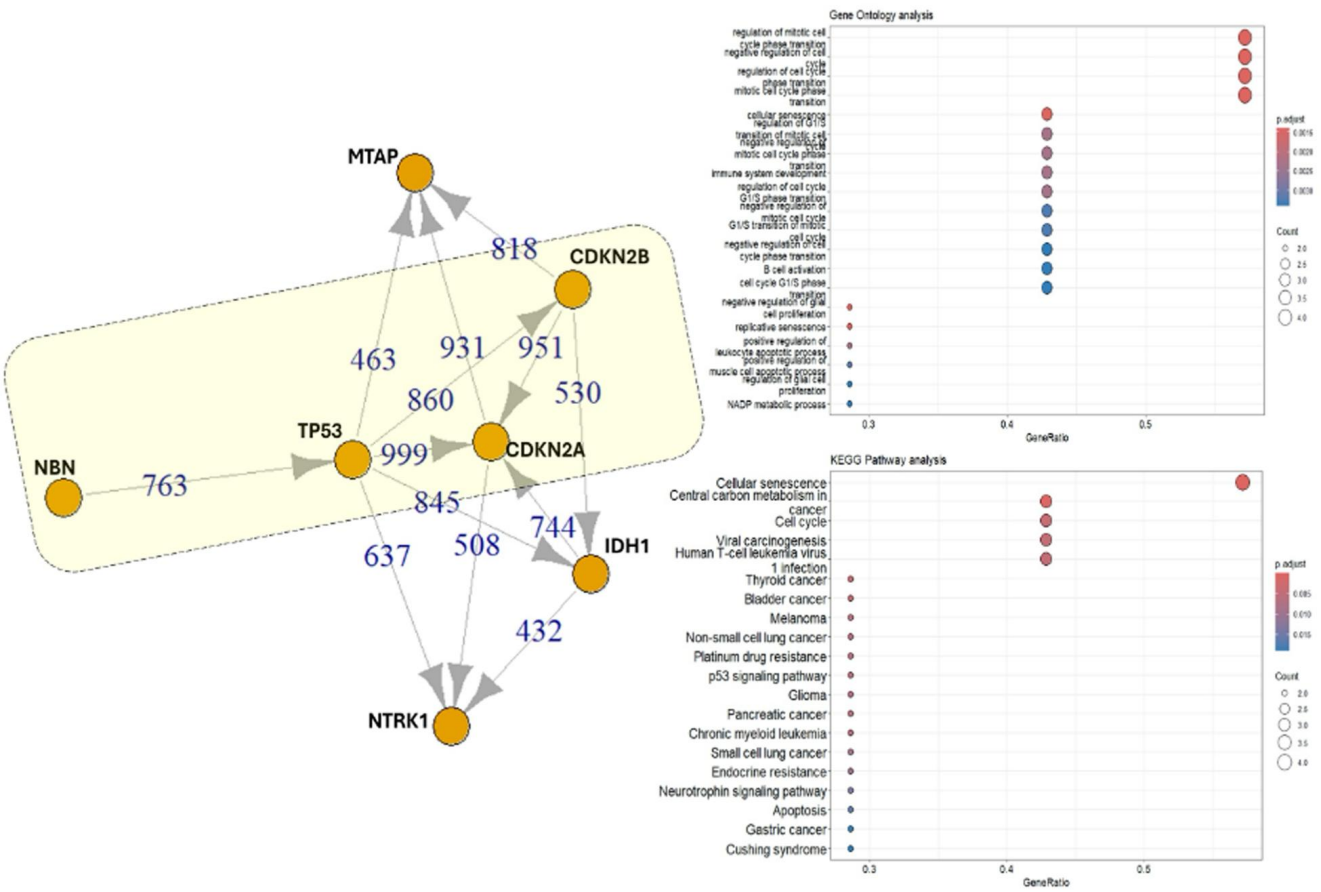
*IDH1* (R132H) and related mutated genes: STRING analysis elucidates interactions between mutated *IDH1* and other mutated genes in patient 1. Four genes exhibit varying degrees of interaction according to STRING, GO, and KEGG analyses (highlighted in the yellow rectangle), with functions in cell cycle regulation and cellular senescence.

Erb-B2 Receptor Tyrosine Kinase 2 (*ERBB2*, R1161Q); Lysine Demethylase 5C (*KDM5C*, P1225S); Lysine Methyltransferase 2A (*KMT2A*, A53V); NFE2 Like BZIP Transcription Factor 2 (*NFE2L2*, A420V); Regulatory Associated Protein of MTOR Complex 1 (*RPTOR*, R997C); Splicing Factor 3b Subunit 1 (*SF3B1*, amplification). According to KEGG analysis, the genes most strongly associated are *CDKN2A*, *CDKN2B*, *NBN*, and *TP53*, all of which are involved in cellular senescence. GO analysis indicates that these genes also play a role in the regulation of mitotic cell division (Figure 2, yellow rectangle).

#### Patient 2

Patient history. No morbid history. Habits, allergies: No; Tobacco: No, Alcohol: No.

Diagnosis: Metastatic biliary malignancy, poorly differentiated carcinoma.

Tissue Biopsy (Sentis):

Microsatellite status: MSS (microsatellite stabilization).

TMB: 0.72 Muts/Mb.

#### Identification of genomic alterations

Isocitrate Dehydrogenase (NADP(+)) (*IDH1*, p. R132L(c.395G>T): 22.99%; CREB Binding Protein (*CREBBP*, p. Q2023*,c.6067C>T): 0.42%; DNA; Nucleotidylexotransferase (*DNTT*, p. R287*,c.859C>T): 0.37%; RecQ Like Helicase (*RECQL*, p. R215*:c.643C>T): 0.37%; B-Raf Proto-Oncogene, Serine/Threonine Kinase (*BRAF*, p. R360*,c.1078C>T): 0.31%; Cyclin D1 (*CCND1*, copy number gain: 3.81); HRas Proto-Oncogene, GTPase (HRAS, copy number gain: 3.97); Tumour protein53 (*TP53*, copy number loss: 0.98).

#### VUS

E74 Like ETS Transcription Factor 3 (*ELF3*); Fc Gamma Receptor IIIa (*FCGR3A*).

**Figure 3.**
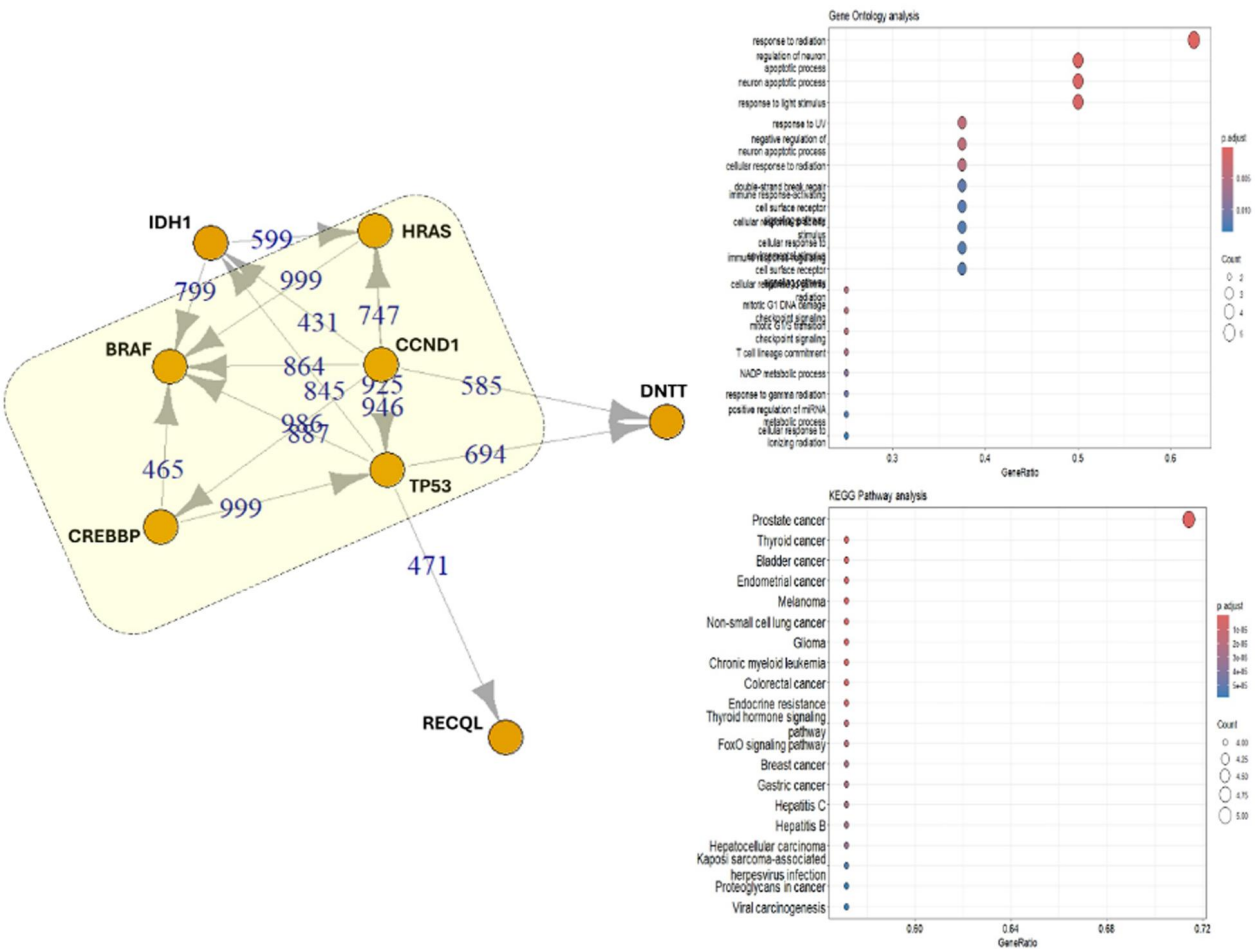
*IDH1* (R132L) and related mutated genes: STRING analysis reveals interactions between mutated *IDH1* and other mutated genes in patient 2. Five mutated proteins identified in the *IDH1*-mutated samples show varying levels of interaction (highlighted in the yellow rectangle). According to STRING, GO, and KEGG analyses, these proteins are involved in the radiation response.

STRING analysis of the genes identified in this patient revealed an association among 5 genes, *CREBBP*, *BRAF*, *CCND1*, *HRAS*, and *TP53*, that are involved in the repair of UV-induced DNA damage, according to GO analysis. Additionally, KEGG analysis indicates that these genes are also implicated in prostate cancer (Figure 3, yellow rectangle).

#### Patient 3

Patient’s medical history: Allergies: No, Tobacco: No; Alcohol: No.

Diagnosis: Metastatic pancreatic NET, well differentiated G3.

3.- Tissue biopsy, 80% tumour cells.

Microsatellite status: MSS.

TMB: Low (1,26).

#### Identification of genomic alterations

Isocitrate Dehydrogenase (NADP (+)) (*IDH1*, p. R132C), VAF 27%, gain of function. Homologous recombination, RAD52 Homolog, DNA Repair Protein (*RAD52*, p. E130K) VAF 46%.

**Figure 4.**
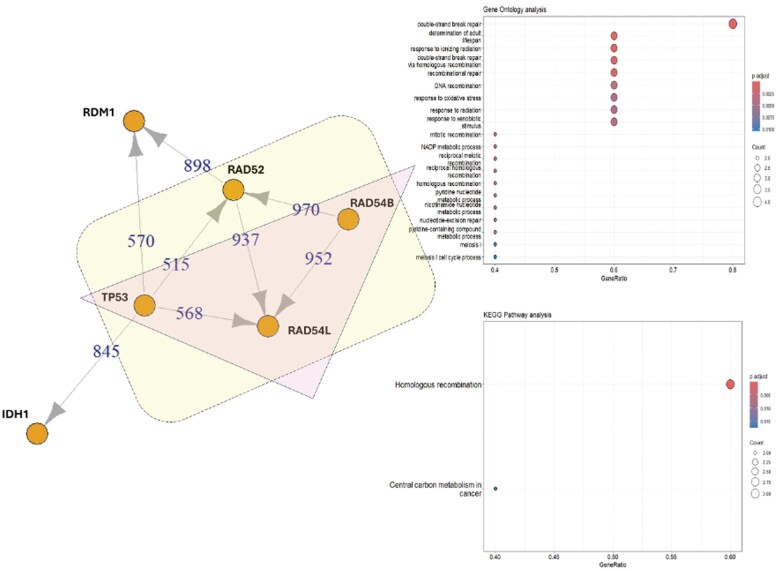
*IDH1* (R132C) and related mutated genes: STRING analysis elucidated an association between mutated *IDH1* and other mutated genes. A cluster of 4 proteins (yellow rectangle) and another cluster of 3 proteins (pink triangles) are highlighted. All proteins exhibited activity in homologous recombination and DNA repair according to STRING, GO, and KEGG analyses.

In this context, although *TP53* was not identified in the NGS analyses, if RAD52 were present, which together with other related proteins such as RAD54B, RAD54L, and TP53 (yellow box), it would constitute a crucial protein involved in DNA double-strand break (DSB) repair and homologous recombination (Figure 4, yellow box). RAD52 binds to single-stranded DNA termini and facilitates the annealing of complementary DNA strands, thereby mediating essential DNA-DNA interactions. TP53-binding protein 1 functions as a DSB repair protein involved in the response to DNA damage and telomere dynamics (Figure 4, yellow box). On the other hand, 3 other genes, like *RAD54L*, *RAD54B*, and *TP53*, are involved in the response to ionizing radiation, oxidative stress, and xenobiotics (Figure 4; pink triangle).

#### Patient 4

Diagnoses: Cholangiocarcinoma.

Biomarker Findings (BGI SENTIS):

Microsatellite status: MSS (microsatellite stabilization).

TMB: 2.15 Muts/Mb.

#### Identification of genomic alterations

*IDH1* (p.R132C(c.394C>T); *PBRM1* (p. E264Mfs*20(c.788_789dupAT); *DNMT3A* (p.Q356*(c.1066C>T).

#### VUS

*MUC16, BAP1, CHEK2, TRRAP, PPM1D, PTPRS, GLI1, PIK3R2, DNMT3*.

**Figure 5.**
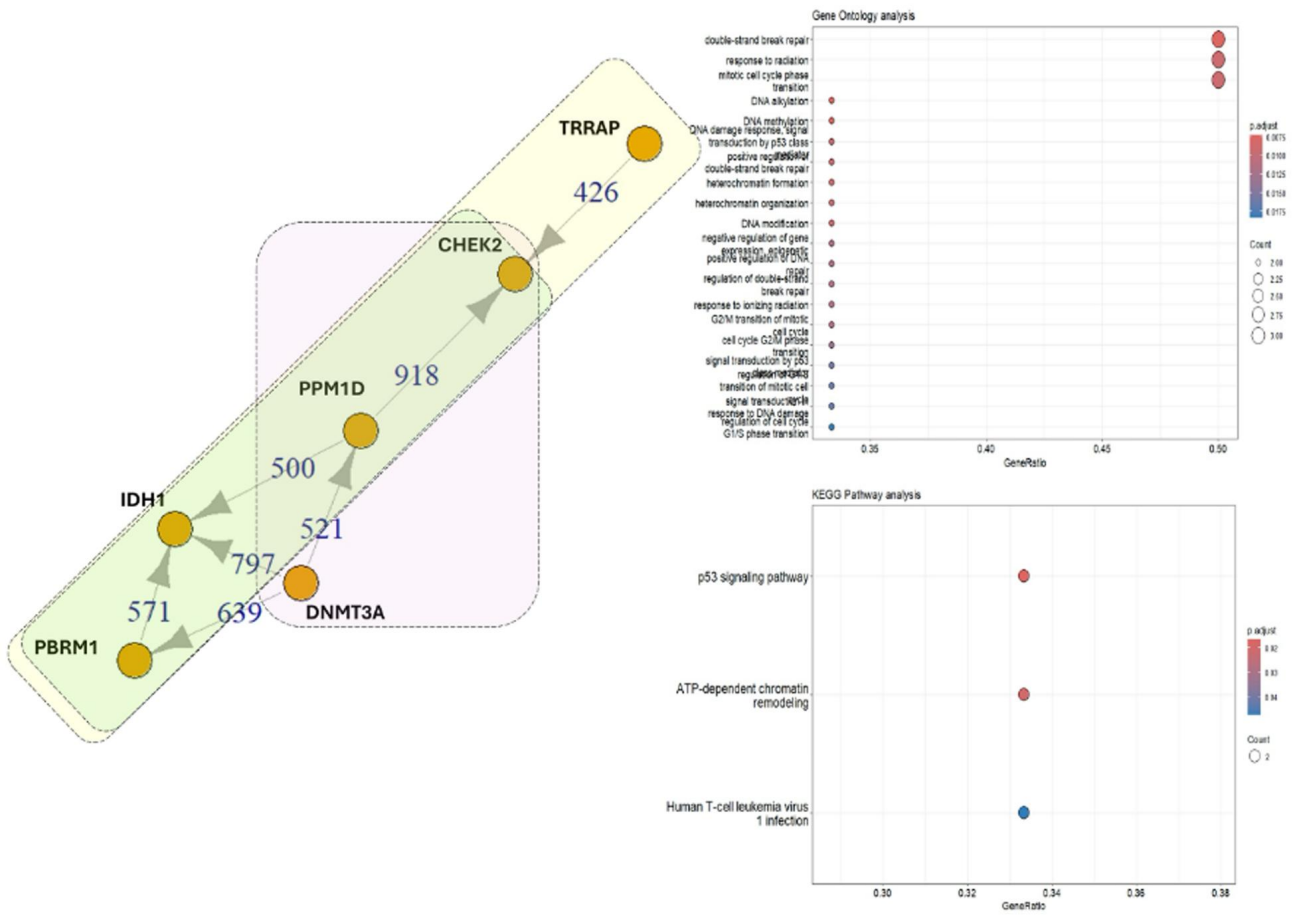
*IDH1* (R132C) and related mutated genes: STRING analysis elucidated interactions between mutated *IDH1* and other mutated genes in patient 4. Three groups of associated proteins were identified: the first group comprises PBRM1, CHEK2, and TRRAP (yellow rectangle); the second group consists of DNMT3A, CHEK2, and PPM1D (pink rectangle); and the third group encompasses PBRM1, PPM1D, and CHEK2 (light green rectangle). All mutated proteins identified in the *IDH1*-mutated samples demonstrated involvement in DNA double-strand repair, response to radiation, and cell cycle regulation according to STRING, GO, and KEGG analyses.

In patient 4, no *TP53* mutation was detected; however, a variant of uncertain significance in *PPM1D*, which negatively regulates TP53 expression, was identified. The STRING and GO analyses revealed a series of associations among mutated genes found in this patient: group 1: PBRM1, CHEK2, and TRRAP (yellow rectangle); group 2: DNMT3A, CHEK2, and PPM1D (pink box), and another group where PBRM1, PPM1D, and CHEK2 are related (light green box). All these mutations, which play a crucial role in checkpoint control and activation of DNA repair in response to DSBs, were also observed. Furthermore, a mutation in *DNMT3A*, which is responsible for DNA methylation, was identified, along with *TRRAP*, which is involved in chromatin complexes with acetyltransferase activity that facilitates epigenetic transcription (Figure 5).

## Discussion

This case review analyses 4 cases involving Chilean patients with a mutation in *IDH1* with different primary malignancies: 1 recurrent oligodendroglioma, 2 malignant biliary tumours, and lastly, a G3 well-differentiated pancreatic neuroendocrine tumour (PanNET, NET G3). Genomic analyses by NGS were conducted to identify cancer-related gene variants in tumour tissue or plasma ctDNA to assess if a more personalized treatment approach would be feasible for these cases. Of note, a significant characteristic of these patients with an *IDH1* mutation was their limited response to conventional or standard chemotherapy or other therapeutic agents and rapid disease progression, highlighting the need to investigate alternative therapeutic strategies for patients harbouring mutation in these genes.^25–27^

Analyses conducted on large cohorts of patients, encompassing both mu/*IDH1* and wild-type/*IDH1* (wt/*IDH1*). Across the entire sample, investigators reported a high incidence of genomic alterations in *CDKN2A, ARID1A, CDKN2B, PBRM1, KRAS/NRAS, BAP1, TP53*, and *FGFR2*, with alterations occurring in ≥10% of patients.^25^ In this research, the genomes of patients with cholangiocarcinoma’s, comprising 152 intrahepatic and 43 extrahepatic cases, from Caucasian, Asian, and African American populations, revealed that the most prevalent mutations were in *IDH1, TP53, ARID1A, BAP1, KRAS, PBRM1, SMAD4*, and *ATM*.^28^^,^^29^ A recent analysis of Chinese patients with cholangiocarcinoma identified *TP53, KRAS, ARID1A, IDH1, SMAD4, FGFR2, BAP1*, and *CDKN2A* as the most frequently mutated genes.^30^ Another key point highlighted in this report was the role of TP53 mutations. Alterations in *TP53* have been shown to negatively affect prognosis in both mutIDH1 and wild-type IDH1 found in patients.^31^ Additionally, *KRAS* and *PIK3CA* alterations were associated with a poorer prognosis in mut/*IDH1* patients, but not in wt/*IDH1* patients. *TP53* mutations have been identified as negative prognostic factors for progression-free survival and overall survival in patients receiving first-line treatment.^25^^,^^32^

STRING analysis was conducted to elucidate the relationships between these mutated genes, revealing mutations in genes involved in DNA repair, including Nibrin (NBN), RAD52 Homolog, DNA Repair Protein (RAD52), Cyclin Dependent Kinase Inhibitor 2 (CDKN2A), RecQ Like Helicase (RECQL), and Checkpoint Kinase 2 (CHEK2). The analysis also identified mutations in genes related to epigenetic modifications, including Lysine Demethylase 5C (KDM5C), DNA Methyltransferase 3 Alpha (DNMT3A), and Tet Methylcytosine Dioxygenase 2 (TET2), as well as in TP53 and Protein Phosphatase, Mg2^+^/Mn2^+^ Dependent 1D (PPM1D), which are involved in the negative regulation of TP53 expression and confer resistance to chemotherapy. These mutations, observed in our case review, collectively indicate a highly unfavourable prognosis for these individuals.

Cancer is a multifaceted and complex disease. In this context, *IDH1* functions as an oncogene, and the production of D-2HG by mutated IDH1 disrupts multiple processes and signalling pathways, including cell proliferation, apoptosis, migration, and drug resistance. This has prompted extensive research and clinical application of *IDH1* inhibitors, which are currently under evaluation in clinical trials and preclinical studies, with diverse outcomes.^33^

## References

[1] Ward PS , Lu C, Cross JR, et al The potential for isocitrate dehydrogenase mutations to produce 2-hydroxyglutarate depends on allele specificity and subcellular compartmentalization. J Biol Chem. 2013;288:3804-3815. 23264629 10.1074/jbc.M112.435495 PMC3567635

[2] Shen D , Zhang J, Yuan K, et al Landscape of IDH1/2 mutations in Chinese patients with solid tumors: a pan-cancer analysis. Mol Genet Genomic Med. 2021;9:e1697. 34145795 10.1002/mgg3.1697 PMC8404228

[3] Reitman ZJ , Yan H. Isocitrate dehydrogenase 1 and 2 mutations in cancer: alterations at a crossroads of cellular metabolism. J Natl Cancer Inst. 2010;102:932-941. 20513808 10.1093/jnci/djq187 PMC2897878

[4] M Gagné L , Boulay K, Topisirovic I, Huot MÉ, Mallette FA. Oncogenic activities of IDH1/2 mutations: from epigenetics to cellular signaling. Trends Cell Biol. 2017;27:738-752. 28711227 10.1016/j.tcb.2017.06.002

[5] Yang B , Zhong C, Peng Y, Lai Z, Ding J. Molecular mechanisms of “off-on switch” of activities of human IDH1 by tumor-associated mutation R132H. Cell Res. 2010;20:1188-1200. 20975740 10.1038/cr.2010.145

[6] Ward PS , Cross JR, Lu C, et al Identification of additional IDH mutations associated with oncometabolite R(-)-2-hydroxyglutarate production. Oncogene. 2012;31:2491-2498. 21996744 10.1038/onc.2011.416 PMC3271133

[7] Golub D , Iyengar N, Dogra S, et al Mutant isocitrate dehydrogenase inhibitors as targeted cancer therapeutics. Front Oncol. 2019;9:417. 31165048 10.3389/fonc.2019.00417 PMC6534082

[8] Kim HJ , Choi BY, Keum YS. Identification of a new selective chemical inhibitor of mutant isocitrate dehydrogenase-1. J Cancer Prev. 2015;20:78-83. 25853107 10.15430/JCP.2015.20.1.78 PMC4384718

[9] Kim NI , Noh M-G, Kim J-H, et al Frequency and prognostic value of IDH mutations in korean patients with cholangiocarcinoma. Front Oncol. 2020;10:1514. 33014795 10.3389/fonc.2020.01514 PMC7461833

[10] Xu W , Yang H, Liu Y, et al Oncometabolite 2-hydroxyglutarate is a competitive inhibitor of α-ketoglutarate-dependent dioxygenases. Cancer Cell. 2011;19:17-30. 21251613 10.1016/j.ccr.2010.12.014 PMC3229304

[11] Chaturvedi A , Araujo Cruz MM, Jyotsana N, et al Enantiomer-specific and paracrine leukemogenicity of mutant IDH metabolite 2-hydroxyglutarate. Leukemia. 2016;30:1708-1715. 27063596 10.1038/leu.2016.71 PMC5298178

[12] Carbonneau M , Gagné LM, Lalonde M-E, et al The oncometabolite 2-hydroxyglutarate activates the mTOR signalling pathway. Nat Commun. 2016;7:12700. 27624942 10.1038/ncomms12700 PMC5027283

[13] Sasaki M , Knobbe CB, Itsumi M, et al D-2-hydroxyglutarate produced by mutant IDH1 perturbs collagen maturation and basement membrane function. Genes Dev. 2012;26:2038-2049. 22925884 10.1101/gad.198200.112 PMC3444730

[14] Nytko KJ , Spielmann P, Camenisch G, Wenger RH, Stiehl DP. Regulated function of the prolyl-4-hydroxylase domain (PHD) oxygen sensor proteins. Antioxid Redox Signal. 2007;9:1329-1338. 17627474 10.1089/ars.2007.1683

[15] Zhao S , Lin Y, Xu W, et al Glioma-derived mutations in IDH1 dominantly inhibit IDH1 catalytic activity and induce HIF-1alpha. Science. 2009;324:261-265. 19359588 10.1126/science.1170944 PMC3251015

[16] Shafei MA , Flemban A, Daly C, et al Differential expression of the BCAT isoforms between breast cancer subtypes. Breast Cancer. 2021;28:592-607. 33367952 10.1007/s12282-020-01197-7 PMC8065012

[17] Bunse L , Pusch S, Bunse T, et al Suppression of antitumor T cell immunity by the oncometabolite (R)-2-hydroxyglutarate. Nat Med. 2018;24:1192-1203. 29988124 10.1038/s41591-018-0095-6

[18] Zhang Y , Wu H, Yang F, et al Prognostic value of the expression of DNA repair-related biomarkers mediated by alcohol in gastric cancer patients. Am J Pathol. 2018;188:367-377. 29331492 10.1016/j.ajpath.2017.10.010 PMC5974541

[19] Galluzzi L , Kroemer G. Potent immunosuppressive effects of the oncometabolite *R* -2-hydroxyglutarate. OncoImmunology. 2018;7:e1528815. 30524910 10.1080/2162402X.2018.1528815 PMC6279319

[20] Notarangelo G , Spinelli JB, Perez EM, et al Oncometabolite d-2HG alters T cell metabolism to impair CD8+ T cell function. Science. 2022;377:1519-1529. 36173860 10.1126/science.abj5104 PMC9629749

[21] Friedrich M , Sankowski R, Bunse L, et al Tryptophan metabolism drives dynamic immunosuppressive myeloid states in IDH-mutant gliomas. Nat Cancer. 2021;2:723-740. 35121943 10.1038/s43018-021-00201-z

[22] Röver LK , Gevensleben H, Dietrich J, et al PD-1 (PDCD1) promoter methylation is a prognostic factor in patients with diffuse lower-grade gliomas harboring isocitrate dehydrogenase (IDH) mutations. EBioMedicine. 2018;28:97-104. 29396294 10.1016/j.ebiom.2018.01.016 PMC5835568

[23] Kelly AD , Kroeger H, Yamazaki J, et al A CpG island methylator phenotype in acute myeloid leukemia independent of IDH mutations and associated with a favorable outcome. Leukemia. 2017;31:2011-2019. 28074068 10.1038/leu.2017.12 PMC5537054

[24] Hill VK , Shinawi T, Ricketts CJ, et al Stability of the CpG island methylator phenotype during glioma progression and identification of methylated loci in secondary glioblastomas. BMC Cancer. 2014;14:506. 25012071 10.1186/1471-2407-14-506 PMC4227105

[25] Rimini M , Fabregat-Franco C, Burgio V, et al Molecular profile and its clinical impact of IDH1 mutated versus IDH1 wild type intrahepatic cholangiocarcinoma. Sci Rep. 2022;12:18775. 36335135 10.1038/s41598-022-22543-z PMC9637171

[26] Boscoe AN , Rolland C, Kelley RK. Frequency and prognostic significance of isocitrate dehydrogenase 1 mutations in cholangiocarcinoma: a systematic literature review. J Gastrointest Oncol. 2019;10:751-765. 31392056 10.21037/jgo.2019.03.10 PMC6657309

[27] Acher AW , Paro A, Elfadaly A, Tsilimigras D, Pawlik TM. Intrahepatic cholangiocarcinoma: a summative review of biomarkers and targeted therapies. Cancers. 2021;13:5169. 34680318 10.3390/cancers13205169 PMC8533913

[28] Churi CR , Shroff R, Wang Y, et al Mutation profiling in cholangiocarcinoma: prognostic and therapeutic implications. PloS One. 2014;9:e115383. 25536104 10.1371/journal.pone.0115383 PMC4275227

[29] Lowery MA , Ptashkin R, Jordan E, et al Comprehensive molecular profiling of intrahepatic and extrahepatic cholangiocarcinomas: potential targets for intervention. Clin Cancer Res Off J Am Assoc Cancer Res. 2018;24:4154-4161. 10.1158/1078-0432.CCR-18-0078 PMC6642361 29848569

[30] Jiang G , Zhang W, Wang T, et al Characteristics of genomic alterations in Chinese cholangiocarcinoma patients. Jpn J Clin Oncol. 2020;50:1117-1125. 32533190 10.1093/jjco/hyaa088

[31] Chao J , Wang S, Wang H, et al Real-world cohort study of PD-1 blockade plus lenvatinib for advanced intrahepatic cholangiocarcinoma: effectiveness, safety, and biomarker analysis. Cancer Immunol Immunother CII. 2023;72:3717-3726. 37787790 10.1007/s00262-023-03523-2 PMC10991235

[32] Zhu AX , Borger DR, Kim Y, et al Genomic profiling of intrahepatic cholangiocarcinoma: refining prognosis and identifying therapeutic targets. Ann Surg Oncol. 2014;21:3827-3834. 24889489 10.1245/s10434-014-3828-x PMC4324507

[33] Liu Y , Shi Y, Han R, et al Signaling pathways of oxidative stress response: the potential therapeutic targets in gastric cancer. Front Immunol. 2023;14:1139589. 37143652 10.3389/fimmu.2023.1139589 PMC10151477

